# Maturation of dendritic cells by pullulan promotes anti-cancer effect

**DOI:** 10.18632/oncotarget.10183

**Published:** 2016-06-20

**Authors:** Wei Zhang, Xiaoqian Yu, Minseok Kwak, Li Xu, LiJun Zhang, Qing Yu, Jun-O Jin

**Affiliations:** ^1^ Shanghai Public Health Clinical Center, Shanghai Medical College, Fudan University, Shanghai, China; ^2^ Periodontal Department, Peking University School and Hospital of Stomatology, Beijing, China; ^3^ Department of Chemistry, Pukyong National University, Busan, South Korea; ^4^ Department of Immunology and Infectious Diseases, The Forsyth Institute, Cambridge, MA, USA; ^5^ Department of Oral Medicine, Infection and Immunity, Harvard School of Dental Medicine, Boston, MA, USA

**Keywords:** pullulan, adjuvant, dendritic cell, immunotherapy, anti-cancer

## Abstract

Previous studies have demonstrated that pullulan, a polysaccharide purified from *Aureobasidium pullulans*, has immune-stimulatory effects on T and B cells. Moreover, pullulan has been used as a carrier in the delivery of the antigen (Ag) peptide to lymphoid tissues. However, the *in vivo* effect of pullulan on dendritic cells (DC) has not been well characterized. In this study, we assessed the effect of pullulan on DC activation and anti-cancer immunity. The results showed that the pullulan treatment up-regulated co-stimulatory molecule expression and enhanced pro-inflammatory cytokine production in bone marrow-derived DCs (BMDC) *in vitro* and in spleen DCs *in vivo*. Moreover, the combination of ovalbumin (OVA) and pullulan induced OVA antigen-specific T cell activations *in vivo*. In tumor-bearing mice, pullulan induced the maturation of DCs in spleen and tumor draining lymph node (drLN), and promoted the OVA-specific T cell activation and migration of the T cells into the tumor. In addition, the combination of OVA and pullulan inhibited B16-OVA tumor growth and liver metastasis. The combination of tyrosinase-related protein 2 (TRP2) peptide and pullulan treatment also suppressed B16 melanoma growth. Thus, the results demonstrated that pullulan enhanced DC maturation and function, and it acted as an adjuvant in promoting Ag-specific immune responses in mice. Thus, pullulan could be a new and useful adjuvant for use in therapeutic cancer vaccines.

## INTRODUCTION

In recent research on the development of therapeutic agents for cancer, the trend has been to search for candidates among natural products because they have relatively low or tolerable toxicity [1]. Over decades, extensive previous studies have demonstrated the effects of some natural products on immune cell functions and responses in the cells of both humans and mice [1–4]. Pullulan, a polysaccharide derived from the yeast-like fungus *Aureobasidium pullulans*, is an α-glucan that consists mainly of maltotriose units joined in α-1, 6 glycosidic linkages [5, 6]. A previous study demonstrated that pullulan induced the production of pro-inflammatory cytokines, including IL-1β, IL-6, IL-8 and TNF-α, in human whole blood cultures [7]. Furthermore, cholesteryl-group-bearing pullulan (CHP), which forms physically cross-linked nanogels by self-assembly in water, was used as a carrier in drug-delivery systems [8–11]. In addition, antigen (Ag) protein delivered by CHP in the absence of additional adjuvant stimulated the nasal immune system and exhibited intranasal vaccine activity [12]. Although many reports indicated that pullulan is useful for drug delivery without adjuvant, the *in vivo* effect of pullulan on immune responses, especially its potential effect as an adjuvant for anti-tumor immune responses, has not been fully investigated.

In the treatment of cancer, immunotherapy aims to induce cancer Ag-specific immune responses that lead to the killing of cancer cells [13, 14]. To achieve this effect, the activation of cytotoxic T lymphocytes (CTL) and effector T helper (Th) cells specific to cancer Ags is critical [13, 15]. However, Ag presenting cells (APC), such as dendritic cells (DC) and macrophages, do not efficiently present cancer Ags via major histocompatibility complex (MHC) molecules, which causes ineffective CTL and Th immune responses against cancer cells [13–15]. Moreover, the cancer microenvironment suppresses inflammatory immune responses by several mechanisms, such as promoting immune tolerance to cancer Ags [16]. Therefore, in cancer immunotherapy, adjuvant is required to overcome immune suppression and induce optimal APC activation, leading to the efficient activation of cancer Ag-specific T cells, which is essential for effective anti-cancer immunity.

An ideally effective adjuvant promotes the maturation and activation of DCs, which then migrate to the spleen and lymph nodes and present Ags via MHC molecules to T cells [13, 14, 17, 18]. Different subsets of DCs have different abilities of Ag-presentation and T cell activation. In mouse, conventional DCs (cDC) have two subsets: CD8α^+^CD11c^+^ and CD8α^−^CD11c^+^ cDCs [3, 19, 20]. CD8α^+^CD11c^+^ cDCs are specialized in the cross-presentation of endogenously synthesized Ags through MHC class I to CTL [20, 21], and CD8α^−^CD11c^+^ cDCs have the selective ability to present extracellular Ags directly through MHC class II to CD4 T cells [22]. Therefore, immunotherapy that involves the activation of both CD8α^+^CD11c^+^ and CD8α^−^CD11c^+^ cDCs by cancer Ags combined with an adjuvant may elicit both CTL and Th immune responses against cancer cells, thereby improving the efficacy of the treatment.

Although pullulan has been shown to induce immune activation in human blood cells and promote Ag-specific immune responses, the direct effects of pullulan on spleen cDCs have not been well characterized. The present study was undertaken to test the hypothesis that pullulan is an effective adjuvant because it induces the activation of spleen cDCs and Ag-specific immune responses *in vivo*, therefore promoting anti-cancer immunity. These results may provide important information for the development of a potential new therapeutic strategy to combat cancer.

## RESULTS

### Pullulan promotes activation of mouse DCs

**Figure 1:**
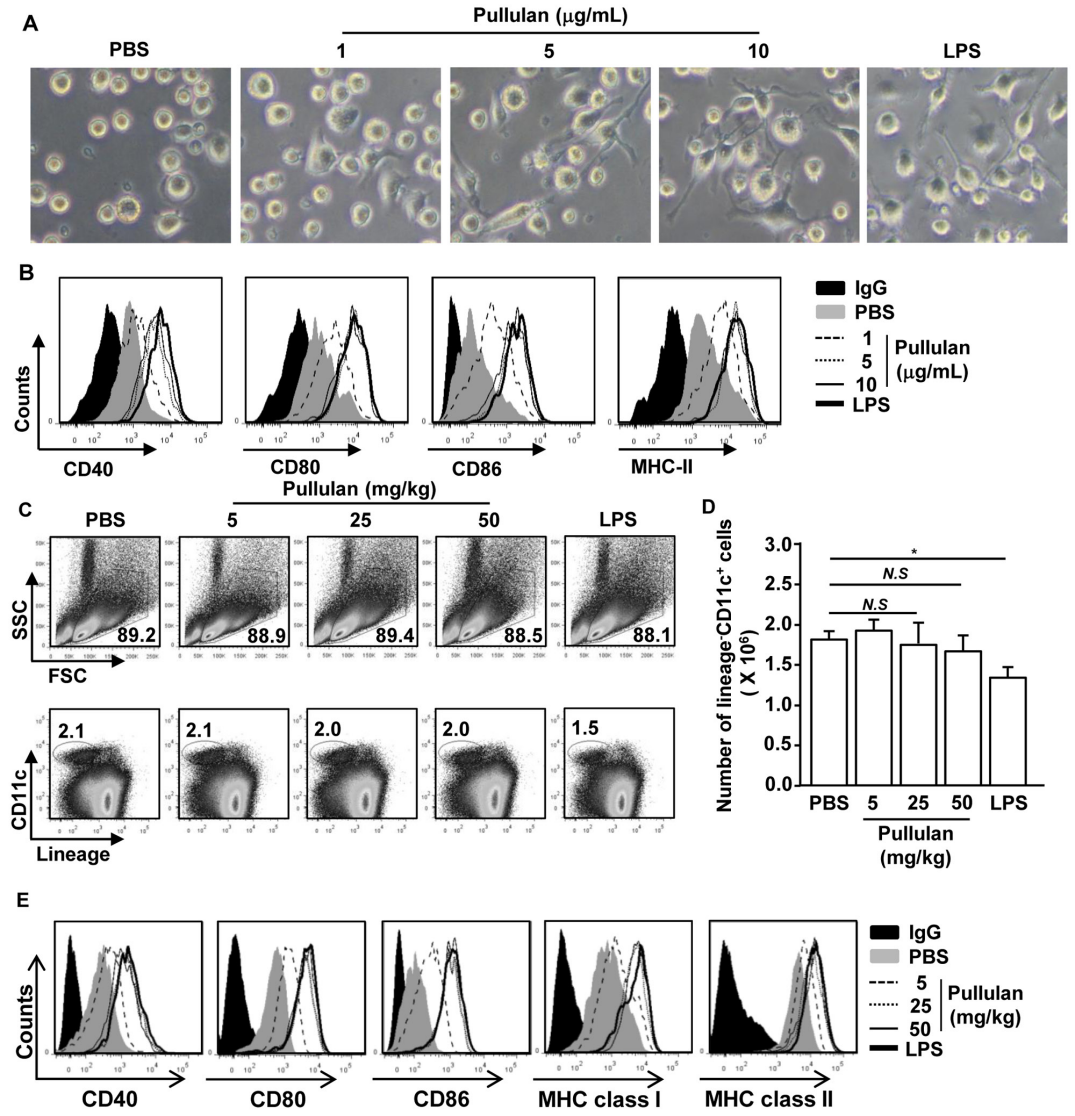
Pullulan induces activation of BMDCs and spleen DCs Bone marrow cells (1 × 10^6^) were incubated with 50 ng/ml GM-CSF and 50 ng/ml IL-4 for 6 days and then stimulated with 1, 5, and 10 μg/mL pullulan or LPS for 24 hours. **A.** Morphological changes were analyzed by microscopy. **B.** Expression of surface co-stimulatory molecules in CD11c^+^ cells. Data are the results of the analyses of 6 independent samples (2 samples per experiment, total 3 independent experiments). C57BL/6 mice were injected intravenously (*i.v.*) with 5, 25, and 50 mg/kg pullulan or 30 μg LPS for 24 hours. **C.** Percentage of lineage^−^CD11c^+^ cDCs was analyzed by flow cytometry. **D.** Absolute numbers of live, lineage^−^CD11c^+^ cells were shown **E.** Expression levels of CD40, CD80, CD86, MHC class I and MHC class II were measured by flow cytometry. All data are representative of or the average of analyses of 6 independent samples (2 mice per experiment, 3 independent experiments); *p < 0.05.

A previous study showed that pullulan induced pro-inflammatory cytokine production in human whole blood cultures [7]. However, the effects of pullulan on DC activation *in vitro* and *in vivo* have not been well characterized. We determined whether pullulan induced the activation of bone marrow-derived DCs (BMDC) *in vitro* and of spleen cDCs *in vivo*. Bone marrow cells from C57BL/6 mice were cultured with granulocyte-macrophage colony-stimulating factor (GM-CSF) and interleukin-4 (IL-4) for 6 days. The cells were further treated with 1, 5, and 10 μg/ml pullulan for 24 hours, using LPS as a positive control. Treatment with pullulan at 5 and 10 μg/ml dramatically promoted the dendritic morphological changes in BMDCs (Figure 1A) and substantially up-regulated the expression of the MHC class II and co-stimulatory molecules CD40, CD80 and CD86 (Figure 1B).

Next, we examined whether pullulan can induce the activation of spleen cDCs *in vivo*. C57BL/6 mice were injected intravenously (*i.v.*) with 5, 25, and 50 mg/kg pullulan, using 30 μg LPS as a positive control. The mice were analyzed for spleen cDC activation 24 hours later. As expected, the LPS treatment led to significant decreases in the proportion and number of spleen cDCs, which were identified as lineage^−^CD11c^+^ cells. In contrast, the pullulan treatment did not alter the proportion and number of spleen cDCs (Figure 1C and 1D). However, similar to LPS, pullulan induced the up-regulation of co-stimulatory molecules and MHC class I and MHC class II molecules at doses of 25 and 50 mg/kg (Figure 1E). Thus, these data indicated that pullulan induced the activation of BMDCs *in vitro* and of spleen cDCs *in vivo*.

### Pullulan promotes the production of pro-inflammatory cytokines from DCs

**Figure 2:**
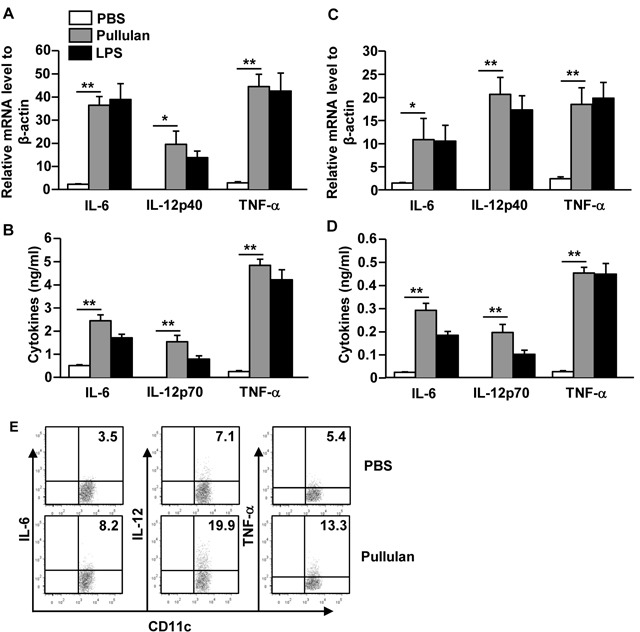
Pullulan induces pro-inflammatory cytokine production on DCs BMDCs were incubated with 5 μg/mL pullulan or LPS for 2 or 24 hours. **A.** mRNA levels of IL-6, IL-12p40 and TNF-α in BMDCs were measured 2 hours after treatment **B.** IL-6, IL-12p70 and TNF-α levels in culture supernatant 24 hours after treatment. **C.** Cytokine mRNA levels in splenocytes were measured 2 hours after 25 mg/kg pullulan and LPS injection. **D.** Cytokine concentrations in sera from pullulan- or LPS-treated mice are shown. **E.** Intracellular cytokine production levels were measured in spleen DCs. All data are representative of or the average of analyses of 6 individual mice each group (2 mice per experiment, 3 independent experiments); *p < 0.05, **p < 0.01.

To determine whether pullulan affects the production of pro-inflammatory cytokines from DCs, we treated BMDCs or C57BL/6 mice with pullulan. The levels of IL-6, IL-12p40, and TNF-α mRNA in pullulan-treated BMDCs were significantly increased compared to the PBS-treated BMDCs (Figure 2A). Consistent with mRNA expression levels, the secretion levels of IL-6, IL-12p70 and TNF-α in a cultured medium of BMDCs treated with pullulan were dramatically increased compared to those treated with PBS (Figure 2B). Moreover, the *in vivo* administration of pullulan led to marked increases in the mRNA levels of IL-6, IL-12p40, and TNF-α in splenocytes (Figure 2C). Furthermore, the serum levels of IL-6, IL-12p70, and TNF-α in the pullulan-treated mice were also significantly higher than in the control mice (Figure 2D).

To measure whether pullulan also stimulated spleen DCs to produce pro-inflammatory cytokines, we examined intracellular cytokine production in pullulan-treated spleen cDCs. As shown in Figure 2E, the pullulan treatment led to marked increases in the percentage of IL-6, IL-12, and TNF-α-producing spleen cDCs compared to the PBS treatment. Thus, these data demonstrated that pullulan induced the activation of BMDCs *in vitro* and of spleen DCs *in vivo*, which was indicated by the up-regulation of co-stimulatory molecules and the production of pro-inflammatory cytokines.

### Pullulan induces Th1 and Tc1 immune responses *in vivo*

**Figure 3:**
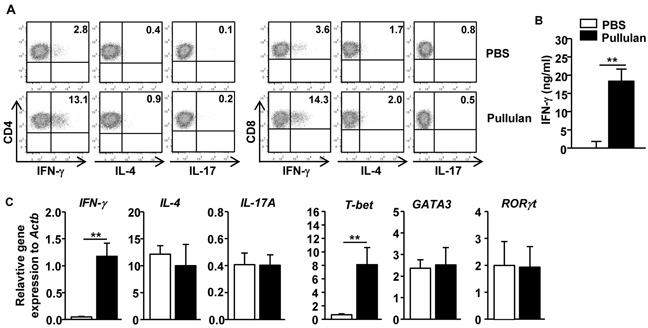
Pullulan promotes IFN-γ-producing CD4 and CD8 T cells *in vivo* C57BL/6 mice were injected *i.v*. with 25 mg/kg pullulan and injected 3 days later with the same amount of pullulan for a further 3 days. **A.** Percentage of IFN-γ, IL-4 and IL-17-producing cells within CD4 and CD8 T cells in spleen were assessed by flow cytometry. **B.** IFN-γ production levels in sera were measured by ELISA. **C.** Expression levels of mRNA were measured from spleen 24 hours after pullulan injection. All data are representative of or the average of analyses of 6 samples from 3 independent experiments; **p < 0.01.

Because pullulan induced spleen cDC activation *in vivo*, we next assessed whether it can therefore promote T cell activation. C57BL/6 mice were injected *i.v.* with pullulan twice, 3 days apart, and then analyzed 3 days after the second injection. The pullulan treatment induced substantial increases in the percentage of IFN-γ-producing CD4 and CD8 T cells in the spleen, whereas it did not affect the percentage of IL-4 and IL-17-producing CD4 and CD8 T cells (Figure 3A). Moreover, the serum concentration of IFN-γ was significantly elevated by the pullulan treatment (Figure 3B). Furthermore, the mRNA levels of IFN-γ and T-bet, which is the critical transcription factor in Th1 and Tc1 cells, were markedly increased in splenocytes by the pullulan treatment, whereas those of GATA3 and RORγt, which are the signature transcription factors for Th2 and Th17, and IL-4 and IL-17A were not changed (Figure 3C). Thus, these data indicated that the administration of pullulan preferentially promoted Th1 and Tc1 responses *in vivo*.

### Pullulan enhances Ag-specific T cell responses

**Figure 4:**
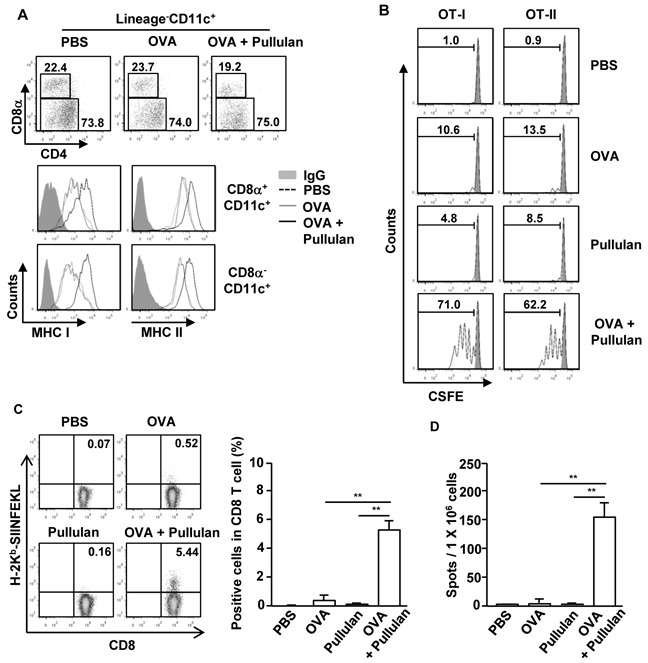
Pullulan promotes antigen presentation and antigen-specific T cell proliferation *in vivo* **A.** C57BL/6 mice were injected with PBS, OVA and combination of OVA and pullulan for 24 hours. The expression levels of MHC class I and II on the gated Lineage^−^CD8α^+^CD11c^+^ and Lineage^−^CD8α^−^CD11c^+^ cDCs in splenocytes from these mice were analyzed. **B.** Purified CD8 T cells from OT-I or CD4 T cells from OT-II mice were labeled with CFSE and transferred into CD45.1 congenic mice; 24 hours later, mice were injected with PBS, OVA, pullulan, and combination of OVA and pullulan. After 3 days of treatment, splenocytes from these mice were stained for CD45.2 to identify the donor OT-I or OT-II cells. The proliferation of these cells was determined by CFSE dilution. **C and D.** C57BL/6 mice were treated *i.v.* with PBS, 50 μg OVA, 25 mg/kg pullulan, and the combination of OVA and pullulan on days 0, 7, and 14. (C) On day 21 after treatment, frequencies and functional activities of OVA (257-264)-specific spleen CD8 T cells were analyzed *ex vivo* by tetramer staining (left panel). Mean percentage of tetramer positive cells in CD8 T cells (right panel). **D.** SIINFEKL-specific IFN-γ-producing cell were analyzed by ELISPOT assay. All data are from analyses of 6 individual mice each group (2 mice per experiment, total 3 independent experiments).

Next, we examined whether pullulan can promote Ag-specific immune responses, including Ag presentation and Ag-specific T cell activation. C57BL/6 mice were injected with PBS, 50 μg ovalbumin (OVA), and the combination of 50 μg OVA and 25 mg/kg pullulan, and then were measured for the expression of MHC class I and II on CD8α^+^CD11c^+^ and CD8α^−^CD11c^+^ spleen cDCs 24 hours later. The combination of OVA and pullulan induced substantial up-regulation of MHC class I and II expression on both CD8α^+^CD11c^+^ and CD8α^−^CD11c^+^ spleen cDCs compared to the OVA treatment alone (Figure 4A). To determine whether the up-regulation of MHC class I and II expression on spleen DCs induced by the combination of OVA and pullulan can subsequently promote OVA-specific OT-I and OT-II T cell proliferation, we transferred CFSE-labeled OT-I and OT-II T cells into CD45.1 congenic mice. Twenty-four hours later, we treated the mice with PBS, 50 μg OVA and the combination of 50 μg OVA and 25 mg/kg pullulan for 3 days before analyzing the cell proliferation by using a dilution of CFSE. The treatment with the combination of OVA and pullulan markedly increased the proliferation of OT-I and OT-II T cells in the spleen compared to OVA or pullulan alone (Figure 4B).

To determine further the adjuvant effect of ascophyllan, C57BL/6 mice were immunized with OVA, pullulan, and the combination of OVA and pullulan on days 0, 7, and 14. On day 21, we measured the OVA-specific CD8 T cell responses by H-2^b^-SIINFEKL tetramer staining and IFN-γ ELISPOT assay. The H-2^b^-SIINFEKL tetramer staining showed that the combination of OVA and pullulan treatment greatly increased the percentage of OVA-specific CD8 cells, whereas OVA or pullulan alone did not promote the increase in OVA-specific CD8 cells (Figure 4C). In addition, the number of IFN-γ-producing CD8 T cells in response to the OVA (257-264) peptide, SIINFEKL, was also substantially increased by the combination of OVA and pullulan treated splenocyte compared to the controls treated with OVA or pullulan alone (Figure 4D). These data suggest that pullulan promotes Ag-specific T cell responses, likely by enhancing Ag presentation by CD8α^+^CD11c^+^ and CD8α^−^CD11c^+^ spleen DCs and thus functions as an immunogenic adjuvant *in vivo*.

### Pullulan induces Ag-specific immune responses in the tumor environment

**Figure 5:**
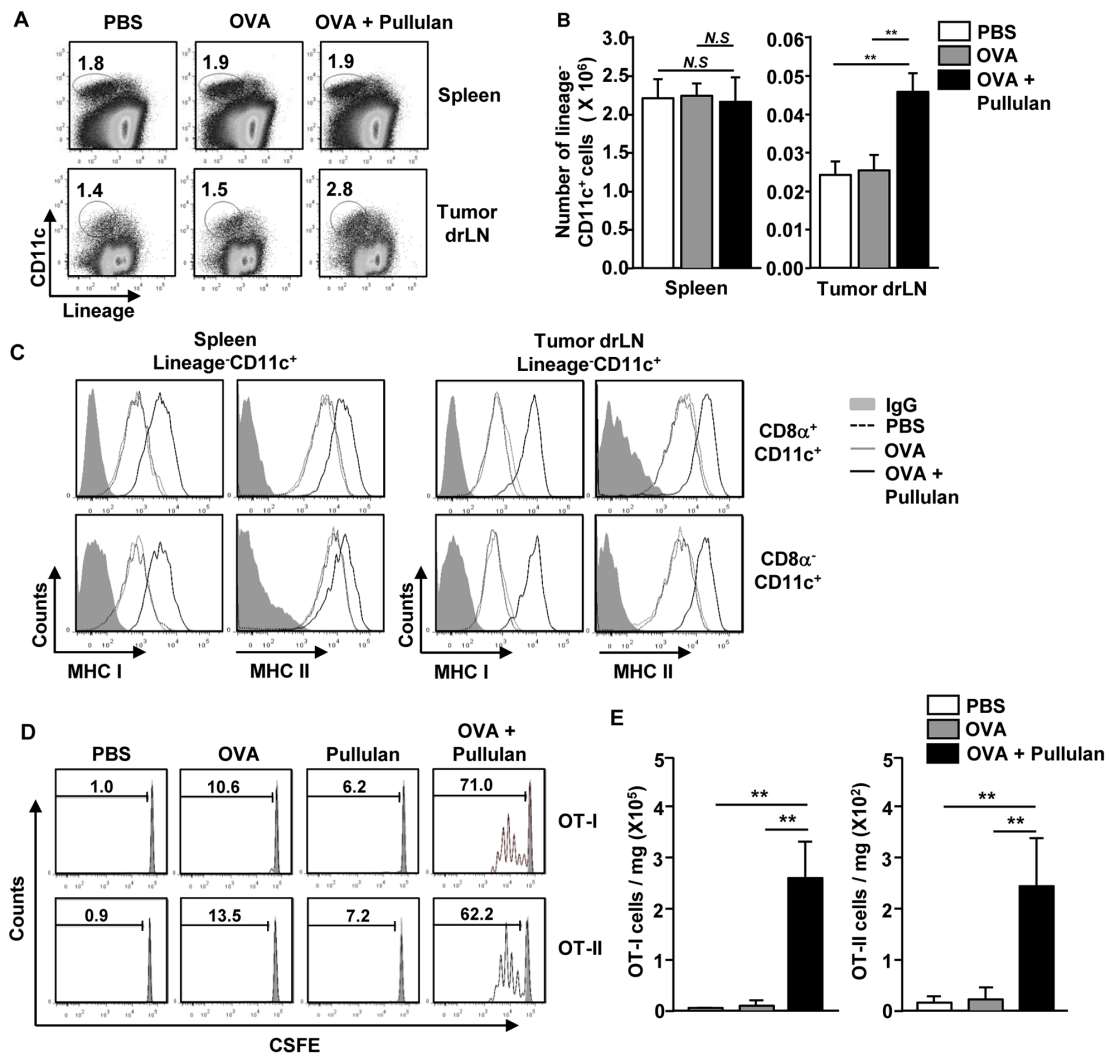
*In vivo* administration of pullulan induces maturation of DCs and antigen specific T cell responses in tumor-bearing mice C57BL/6 mice were injected *subcutaneously* (*s.c.*) with 1 × 10^6^ B16 melanoma cells. When the tumors reached the average volume of 500–1000 mm^3^, the mice were treated with 25 mg/kg pullulan for 24 hours and harvested for spleen and tumor draining lymph node (drLN). (A) Percentages of lineage^−^CD11c^+^ DCs in spleen and tumor drLN were analyzed on a flow cytometry. **B.** Absolute number of lineage^−^CD11c^+^ cells within live cells in spleen (left panel) and tumor drLN (right panel). **C.** Flow cytometric analysis of MHC class I and II expression in gated lineage^−^CD8α^+^CD11c^+^ cells and lineage^−^CD8α^−^CD11c^+^ cells from spleen and tumor drLN. **D.** OT-I or OT-II cells were labeled with CFSE and transferred into B16 tumor-bearing CD45.1 congenic mice, and 24 hours later, mice were injected with PBS, OVA, pullulan, and combination of OVA and pullulan. After 3 days of injection, the proliferation of OT-I and OT-II cells was determined by CFSE dilution. **E.** Absolute number of OT-I (left panel) and OT-II (right panel) cells in the tumor is shown. All data are from analyses of 6 individual mice each group (2 mice per experiment, total 3 independent experiments); **p < 0.01.

Our observation that pullulan enhanced Ag presentation in spleen cDC subsets and promoted Ag-specific T cell activation *in vivo* prompted us to examine whether pullulan can induce similar immune activation in the tumor-bearing mice. C57BL/6 mice were injected subcutaneously (*s.c.*) with 1 × 10^6^ B16 melanoma cells. After 15 days of tumor cell injection, the mice received *i.v.* injections of a combination of OVA and pullulan, and they were analyzed for the activation of cDCs in the spleen and tumor draining lymph node (drLN) 24 hours later. The treatment of tumor-bearing mice with the combination of OVA and pullulan substantially increased the percentage and number of DCs in the tumor drLN, whereas those in the spleen were not altered (Figure 5A and 5B). Moreover, MHC class I and II expression levels on CD8α^+^CD11c^+^ and CD8α^−^CD11c^+^ DCs in the spleen and tumor drLN were considerably up-regulated by the combination of OVA and pullulan, whereas they were not up-regulated by OVA alone (Figure 5C).

Next, we examined whether the combination of OVA and pullulan can induce OVA specific T cell activation in the tumor-bearing mice. We injected *s.c.* 1 × 10^6^ B16-OVA melanoma cells into CD45.1 congenic mice. When the tumors reached the average volume of 500–1000 mm^3^, we transferred the CFSE-labeled OT-I and OT-II T cells into the tumor-bearing CD45.1 congenic mice. After 24 hours, PBS, OVA, and the combination of OVA and pullulan were injected into these mice, which were analyzed 3 days later. The combination of OVA and pullulan induced substantial increases in the proliferation of spleen OT-I and OT-II T cells in the tumor-bearing mice compared to OVA or pullulan alone (Figure 5D). In addition, the combination of OVA and pullulan induced the significant infiltration of OT-I and OT-II T cells in the tumor, whereas OVA alone did not (Figure 5E). Thus, these data suggest that the pullulan treatment promotes Ag-specific immune responses in a tumor environment.

### The combination of OVA and pullulan inhibits B16-OVA tumor growth

**Figure 6:**
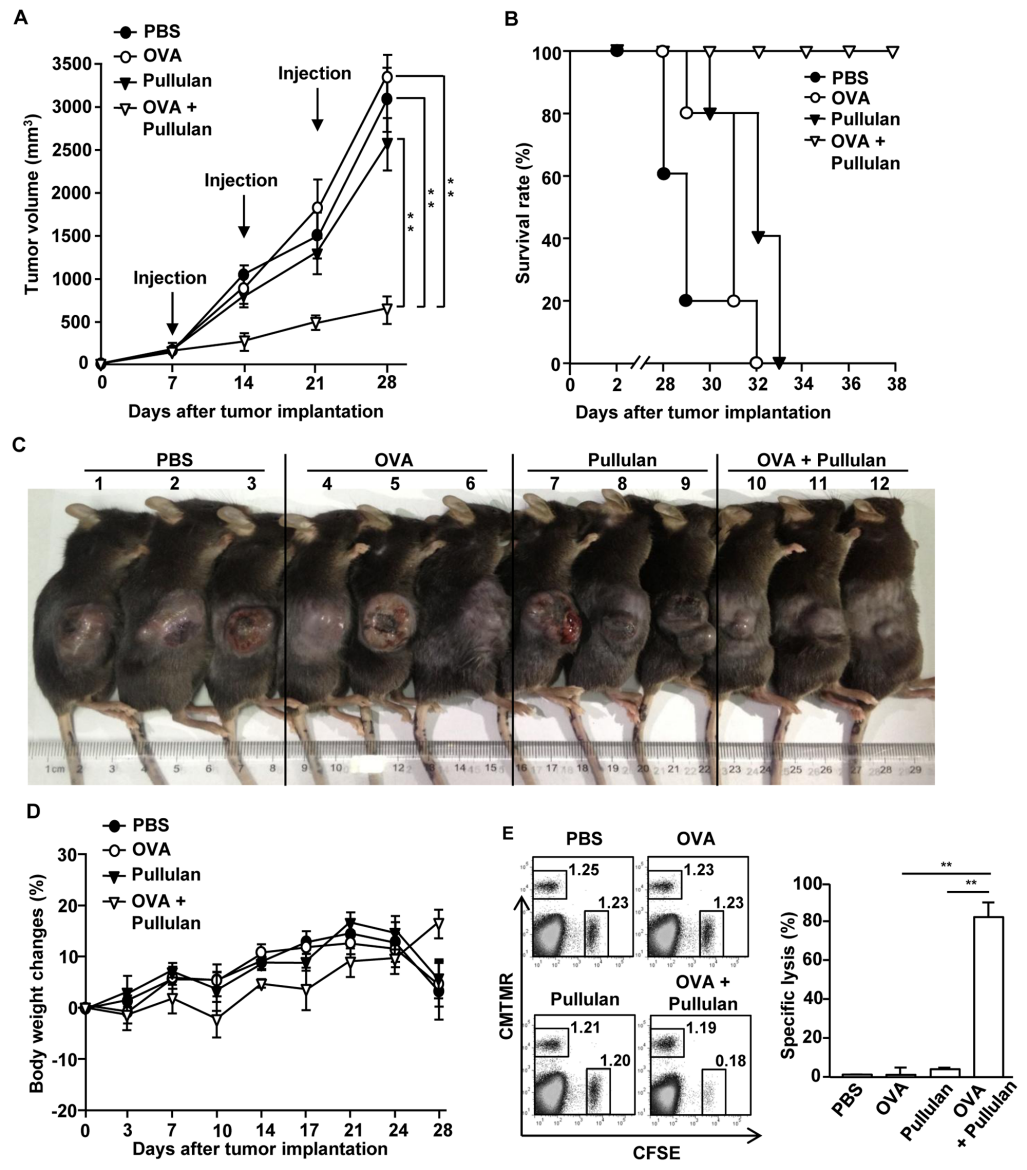
Treatment of combination of OVA and pullulan inhibits B16-OVA tumor cell growth *in vivo* C57BL/6 mice were injected *s.c.* with 1 × 10^6^ B16-OVA cells on right flanks of mice. When the tumors reached the average volume of 30–40 mm^3^, mice received *i.v.* with PBS, 50 μg OVA, 25 mg/kg pullulan, and combination of OVA and pullulan on day 7, 14 and 21. **A.** B16-OVA tumor growth curves in the mice are shown. **B.** Survival rate of mice is shown. Data are representative of or the average of analyses of 5 independent samples (2 or 3 mice per experiment, 2 independent experiments). **, statistically significant values, defined as *P < 0.01* and determined by paired Student's t test, compared with corresponding groups. **C.** Tumor mass in the mice was shown after the mice were sacrificed on day 24 of B16-OVA tumor cell challenge. **D.** Body weight of mice is shown during tumor challenge. **E.** Cytotoxic T lymphocyte (CTL) activity was assessed *in vivo* at 25 days of treatment by adoptively transfer of splenocytes populations labeled with CFSE and loaded with SIINFEK. A control splenocyte population without peptide was labeled with CMTMR. The dot plots show percentages of SIINFEK-loaded CFSE^+^ cells and non-peptide-loaded CMTMR^+^ cells (left panel). Mean percentages of Ag-specific lysis (right panel). Data are from analyses of 5 individual mice each group (2 or 3 mice per experiment, 2 independent experiments); **p < 0.01.

Since the combination of OVA and pullulan enhanced OVA specific immune responses in the tumor-bearing mice, we then examined whether the combination of OVA and pullulan can inhibit B16-OVA tumor growth *in vivo*. C57BL/6 mice were injected *s.c.* with 1 × 10^6^ B16-OVA cells. When the tumors reached the average volume of 30–40 mm^3^, the mice were injected with PBS, 50 μg OVA and the combination of 50 μg OVA and 25 mg/kg pullulan on days 7, 14, and 21 after the tumor challenge. The combination of OVA and pullulan dramatically suppressed B16-OVA tumor growth compared to PBS, OVA, and pullulan alone (Figure 6A). Moreover, the mice treated with the combination of OVA and pullulan survived much longer than those treated with PBS, OVA, and pullulan alone (Figure 6B). As shown in Figure 6C, the sizes of the tumor masses in the mice treated with the combination of OVA and pullulan on day 24 were much smaller than those in the mice injected with OVA and pullulan alone. In addition, the body weight of the combination of OVA and pullulan-treated tumor-bearing mice was gradually increased, but the weight of the OVA- or pullulan-treated mice peaked at day 21 of the tumor challenge and then rapidly decreased (Figure 6D).

We next examined the functional activity of CTLs by using an *in vivo* cytotoxicity assay. C57BL/6 mice were immunized with OVA, pullulan, and the combination of OVA and pullulan on days 0, 7, and 14. On day 21 after the initial immunization, the mice were transferred *i.v.* with CFSE-labeled and SIINFEKL-pulsed splenocytes and CMTMR-labeled and non-peptide-loaded splenocytes from C57BL/6 donor mice and measured for specific cell lysis by flow cytometry. The specific target cell lysis was 80% in the mice immunized with the combination of OVA and pullulan (Figure 6E). In contrast, the mice immunized with OVA or pullulan alone showed no significant killing of OVA-pulsed splenocytes (Figure 6E). Therefore, these data demonstrated that pullulan treatment combined with OVA immunization inhibited B16-OVA tumor growth *in vivo*.

### The combination of OVA and pullulan prevents liver metastasis of B16-OVA melanoma cells

**Figure 7:**
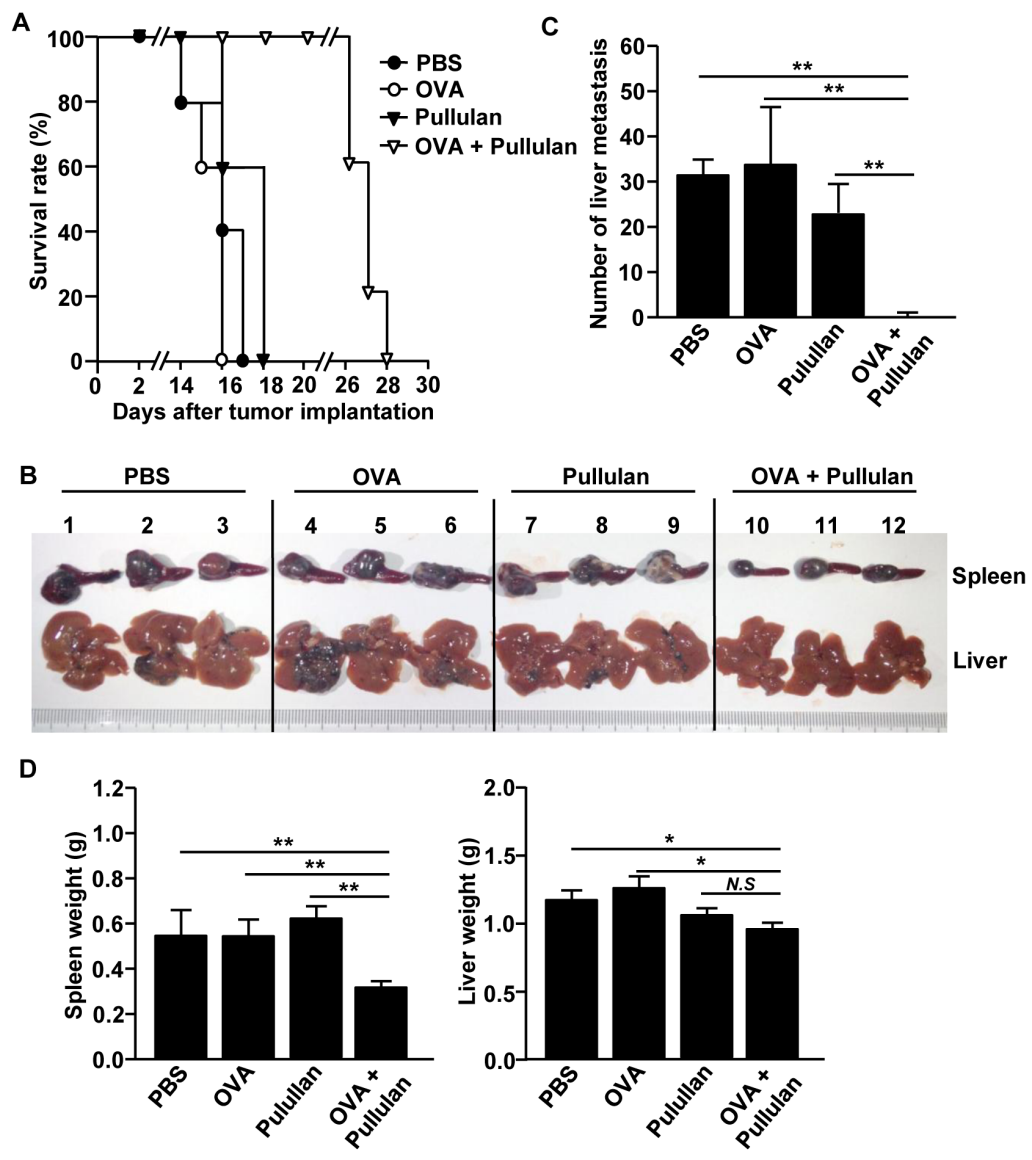
Combination of OVA and pullulan prevent metastasis of B16-OVA melanoma cells into liver C57BL/6 mice were injected *i.v.* with PBS, 50 μg OVA, 25 mg/kg pullulan and combination of OVA and pullulan. Three days after treatment, the mice were inoculated intrasplenically (*i.s*.) with 0.5 × 10^6^ / 50 μl B16-OVA melanoma cells. On days 3 and 6 of tumor challenge, mice received the same amount of treatment. **A.** Survival rate of mice is shown. **B.** Size of tumor mass in spleen and metastasis of B16-OVA cells in liver on day 14 of tumor injection are shown. **C.** Absolute number of B16-OVA metastasis into liver. **D.** Weights of spleen (left panel) and liver (right) are shown. All data are representative of or the average of analyses of 5 independent samples (2 or 3 mice per experiment, total 2 independent experiments); **p < 0.01, * p<0.05.

We next examined whether treatment with the combination of OVA and pullulan can inhibit tumor metastasis in mice. We injected *i.v.* the mice with PBS, 50 μg OVA, 25 mg/kg pullulan, and the combination of OVA and pullulan. After 3 days of treatment, the mice were inoculated intrasplenically (*i.s*.) with 0.5 × 10^6^ B16-OVA melanoma cells and *i.v.-*injected with PBS, OVA, pullulan, and the combination of OVA and pullulan twice, at 3-day intervals. The mice treated with PBS, OVA, and pullulan alone died within 18 days of the tumor challenge. In comparison, the mice treated with the combination of OVA and pullulan started dying on day 26, and all were dead within day 28 of the B16-OVA challenge (Figure 7A). Moreover, the size of the tumor mass in the spleen on day 14 after the B16-OVA challenge was substantially smaller in mice treated with the combination of OVA and pullulan compared to the other treatment groups (Figure 7B). Furthermore, the mice treated with the combination of OVA and pullulan were almost completely protected from the B16-OVA cell invasion of the liver, whereas those treated with PBS, OVA, and pullulan alone showed a significant number of B16-OVA tumor cells in the liver (Figure 7B and 7C). In addition, spleen and liver weight were significantly lighter in the mice treated with the combination of OVA and pullulan than in the other treatment groups (Figure 7D). Collectively, these data suggest that the combination of OVA and pullulan prevented the liver metastasis of B16-OVA melanoma cells.

### The combination of self tumor-associated antigen and pullulan prevents B16 melanoma growth

**Figure 8:**
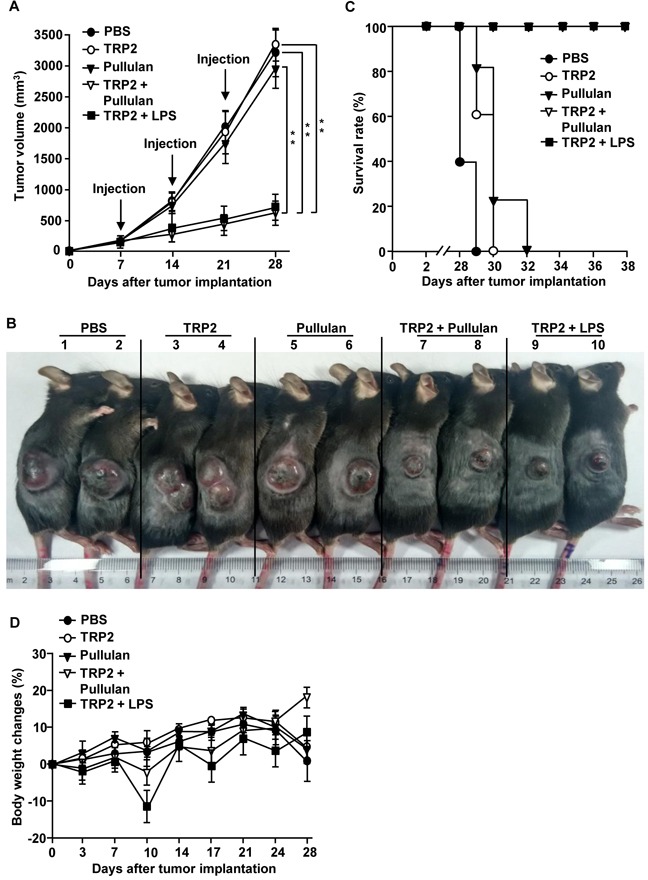
Combination of TRP2 and pullulan inhibits B16 melanoma growth *in vivo* C57BL/6 mice were injected *s.c.* with 1 × 10^6^ B16 cells on the right flanks of mice. When the tumors reached the average volume of 30–40 mm^3^, the mice received *i.v.* with PBS, 50 μg TRP2 peptide, 25 mg/kg pullulan, the combination of TRP2 and pullulan, and the combination of TRP2 and LPS on day 7, 14 and 21. **A.** B16 tumor growth curves in the mice are shown. **B.** Tumor mass in the mice was shown after the mice were sacrificed on day 24 of the B16 tumor cell challenge. **C.** Survival rate of mice is shown. **D.** Body weights of mice are shown during tumor challenge. Data are representative of or the average of analyses of 5 independent samples (2 or 3 mice per experiment, 2 independent experiments). ** Statistically significant values were defined as *P < 0.01,* determined using a paired Student's t test and compared with corresponding groups.

To confirm the adjuvant activity of pullulan in the self tumor-associated antigen, we next examined the combination of tyrosinase-related protein 2 (TRP2) peptide and pullulan in the B16 melanoma tumor-bearing mice. C57BL/6 mice were injected *s.c.* with 1 × 10^6^ B16 cells. When the tumors reached the average volume of 30–40 mm^3^, the mice were injected with PBS, 50 μg TRP2 peptide, 25 mg/kg pullulan, and the combination of 50 μg TRP2 peptide and 25 mg/kg pullulan on day 7, 14, and 21 of the tumor challenge. As the positive control, we also injected the mice with the combination of TRP2 peptide and LPS. The combination of TRP2 peptide and pullulan substantially inhibited B16-OVA tumor growth compared to PBS, TRP2 peptide and pullulan alone (Figure 8A). Moreover, the size of the tumor mass in the mice treated with the combination of TRP2 peptide and pullulan on day 24 was also much smaller than in the mice injected with TRP2 peptide or pullulan alone (Figure 8B). In addition, the mice treated with the combination of TRP2 peptide and pullulan survived much longer than mice injected with TRP2 peptide or pullulan alone (Figure 8C). Furthermore, the pullulan showed almost the same adjuvant activities as LPS in the suppression of tumor growth. Unlike LPS, pullulan did not reduce body weight during the treatment of tumors in the mice (Figure 8D). Thus, these data suggest that pullulan may be safer than LPS and pullulan treatment combined with self tumor-associated Ag can inhibit melanoma tumor growth *in vivo*.

## DISCUSSION

Because of their low toxicity, natural products have recently emerged as promising therapeutic reagents for cancer and infectious diseases [1]. Various polysaccharides, including fucoidan, ascophyllan and λ-carrageenan, have shown immune activation and anti-cancer effects [3, 19, 23, 24]. In this study, we showed that pullulan, a polysaccharide purified from *Aureobasidium pullulans*, promoted the activation of BMDCs *in vitro* and of spleen DCs *in vivo,* and induced anti-cancer immune responses. These findings suggest that pullulan may be a promising new adjuvant for cancer immunotherapy.

Recent studies have shown that pullulan can be used as a carrier for Ag delivery in vaccine development [9, 12, 25]. However, the effects of CHP alone on immune responses have not been well characterized. In this study, we demonstrated that pullulan induced spleen DC activation *in vivo*. Further investigations will be conducted to determine the effects of CHP on spleen DCs and to compare the immune-activating effects of pullulan with CHP.

The maturation and activation of DCs are characterized by the increased expression of co-stimulatory molecules and the production of pro-inflammatory cytokines [13, 14, 18]. DC-based vaccines are considered useful in cancer immunotherapy, and the interaction between activated DCs and cancer Ags is important in the design of vaccines. Most DC-based vaccines employed *in vitro* generated DCs, such as BMDCs and monocyte-derived DCs (MDDCs), which were stimulated by an adjuvant and cancer Ag *in vitro* and then transferred into recipient mice to enhance Ag-specific immunity [26, 27]. However, *in vitro* generated DCs differed in phenotype and function from DCs generated *in vivo* [28]. Moreover, *in vitro* generated DCs may not maintain stability in recipient cancer patients. In contrast, the pullulan treatment induced the up-regulation of co-stimulatory molecules and production of pro-inflammatory cytokines in DCs *in vivo* and induced the activation of both CD8α^+^CD11c^+^ and CD8α^−^CD11c^+^ cDCs in the spleen. Because different DC subsets promote different types of T cell activation [21], the pullulan-induced activation of both CD8α^+^CD11c^+^ and CD8α^−^CD11c^+^ DCs may promote both CTL and Th immune responses. Moreover, we showed that treatment with the combination of OVA and pullulan prevented B16-OVA tumor growth *in vivo*. In addition, the combination of self tumor-associated antigen and pullulan inhibited B16 melanoma growth. Therefore*,* pullulan has the potential to be used as a therapeutic cancer vaccine by *in vivo* administration combined with cancer Ags.

Since the cancer microenvironment suppresses immune activation against cancer Ags, the development of therapeutic cancer vaccines is a challenging task [17, 29, 30]. In cancer microenvironments, DC activation and T cell expansion are limited by immune-suppressing molecules derived from cancer cells[15]. Thus, an effective adjuvant is required as part of an efficient therapeutic cancer vaccine to help circumvent regulatory mechanisms and boost immune responses against cancer Ags [17, 29]. The findings of the present study indicated that pullulan induced the activation of DCs in the spleen and tumor drLN in tumor-bearing mice. Moreover, pullulan exhibited an adjuvant activity of priming OVA Ag-specific T cell responses. Finally, treatment with the combination of OVA and pullulan in tumor-bearing mice successfully inhibited B16-OVA tumor growth, likely because of the enhanced Ag-specific immune activation. Interestingly, the combination of OVA and pullulan increased the number of DCs in the tumor drLN, but not in the spleen, which suggests that pullulan may enhance the migration of DCs to tumor drLN for Ag presentation to T cells. In future studies, we will investigate whether pullulan promotes DC migration to tumor drLN in tumor-bearing mice by using the adaptive transfer of GFP-expressing DCs.

Because metastasis is the major cause of mortality in cancer patients, there is crucial need to prevent tumor metastasis in cancer therapy [31]. Ag-specific immune activation is a powerful mechanism for the prevention of metastasis because Ag-specific T cells can find and eliminate metastasized cancer cells [32]. The results of the present study showed that the combination of OVA and pullulan inhibited liver metastasis of B16-OVA melanoma cells *in vivo*. These data suggest that pullulan-induced OVA-specific T cell responses may be able to kill circulating tumor cells *in vivo*.

In conclusion, our results provide evidence that pullulan is an effective adjuvant that can induce DC and T cell activation and Ag-specific immune responses in tumor-bearing mice. Moreover, treatment with the combination of Ags and pullulan could be used to prevent tumor growth and metastasis in mice. Hence, pullulan may be useful as a new adjuvant in the development of effective therapeutic cancer vaccines.

## MATERIALS AND METHODS

### Mice and cell lines

C57BL/6 mice (6 weeks old), OT-I and OT-II TCR transgenic mice and C57BL/6-Ly5.1 (CD45.1) congenic mice were obtained from the Shanghai Public Health Clinical Center, and kept under pathogen-free conditions. All experiments were carried out under the guidelines of the Institutional Animal Care and Use Committee at the Shanghai Public Health Clinical Center. The protocol was approved by the Committee on the Ethics of Animal Experiments of the Shanghai Public Health Clinical Center (Mouse Protocol Number: SYXK-2010-0098). The mice were sacrificed by CO_2_ inhalation euthanasia, and all efforts were made to minimize their suffering. The murine melanoma cell line B16F10 (ATCC, CRL-6475) expressing OVA (B16-OVA) was cultured in 10% FCS RPMI (Sigma Aldrich, 2 mM glutamine, 1 M HEPES, 100 μg/ml streptomycin and 100 U/ml penicillin, 2 mM 2-mercaptoethanol). All cell lines were cultured at 37°C in a humidified atmosphere of 5% CO_2_ and air.

### Chemicals and cytokines

Pullulan from *Aureobasidium pullulans* and chicken OVA were obtained from Sigma-Aldrich. The pullulan solution was passed through an endotoxin-removal column (Detoxi-gel: Thermo Fisher Scientific), and subsequently filtered through an endotoxin-removal filter (Zetapor Dispo: Wako). The pullulan was freshly prepared before its use in the treatment of the mice. OVA (257-264: SIINFEKL) peptide and TRP2 peptide (SVYDFFVWL) were obtained from Synpeptide (Shanghai).

### Antibodies

Isotype control antibodies (Abs) (IgG1, IgG2a or IgG2b), CD11c (HL3), CD4 (GK1.5), CD8α (YTS169.4), CD40 (3/23), CD80 (16-10A1), CD86 (GL-1), anti-IL-4 (11B11), anti-IL-6 (MP5-20F3) and anti-IL-12/23p40 (C17.8) were obtained from BioLegend; anti-MHC class I (AF6-88.5.3), anti-MHC class II (M5/114.15.2), anti-IFN-γ (XMG1.2), anti-IL-17 (TCC11-18H10.1) and anti-TNF-α (MP6-XT22) were obtained from eBioscience.

### Flow cytometry analysis

The cells were washed with phosphate buffered saline (PBS) containing 0.5% BSA, pre-incubated for 15 min with unlabeled isotype control Abs, and then labeled with fluorescence-conjugated Abs by incubation on ice for 30 min followed by washing with PBS. The cells were analyzed using FACS Aria II (Becton Dickinson) and FlowJo 8.6 software (Tree Star). Cellular debris was excluded from the analysis by forward- and side-scatter gating. Dead cells were further excluded by 7 aminoactinomycin D (7AAD) (BioLegend) staining and gating on the 7AAD-negative population. As the control for nonspecific staining, mAbs, isotype-matched irrelevant, were used.

### *In vitro* BMDC Generation

The initial cultures were prepared as described previously [19, 33]. Bone marrow nucleated cells (1 × 10^6^ cells/mL) were cultured in 5 mL modified RPMI 1640 medium containing 10% FBS in 6 well plates: 50 ng/mL rmGM-CSF plus 50 ng/mL rmIL-4 were added to the medium to support the generation of BMDCs. Unless otherwise stated, the cells were cultured for 6 days at 37°C under 10% CO_2_. The cultured cells were washed twice in fresh medium before the additional experiments were conducted.

### DC analysis

Spleen and tumor drLN DCs were analyzed as described previously [3, 24, 33]. Briefly, the tissues were cut into small fragments and digested by adding 2% fetal bovine serum (FBS) with collagenase for 20 min at room temperature. The cells from the digest were centrifuged to pellets, which were re-suspended in 5 mL of a 1.077 histopaque (Sigma-Aldrich). An additional 5 mL of histopaque and 1 mL of culture medium were layered below and above the cell suspension, respectively, which was then centrifuged at 1700 g for 10 min. The light density fraction (< 1.077 g/cm^3^) was collected and incubated for 20 min with the following FITC-conjugated monoclonal antibodies (mAbs): anti-CD3 (17A2), anti-Thy1.1 (OX-7), anti-B220 (RA3-6B2), anti-Gr-1 (RB68C5), anti-CD49b (DX5) and anti-TER-119 (TER-119). The lineage^−^CD11c^+^ cells were defined as cDCs, which were further divided into CD8α^+^CD11c^+^ and CD8α^−^CD11c^+^ cDCs. The analysis was carried out using a FACS Aria II (Becton Dickinson).

### *Ex vivo* T cell stimulation and intracellular cytokine staining

As described previously [34], the single cells prepared from spleen were stimulated *in vitro* for 4 hours with phorbol 12-myristate 13-acetate (PMA) (50 ng/ml; Calbiochem) and ionomycin (1 μM; Calbiochem), Monensin Solution (Biolegend) was added during the final 2 hours. In the intracellular cytokine staining, the cells first were stained for surface molecules, fixed and permeabilized with Cytofix/Cytoperm buffer (eBioscience), and subsequently incubated with anti-cytokine antibodies in Perm/Wash buffer (eBioscience) for 30 min. Control staining with isotype control IgGs was performed in all experiments.

### ELISA

IL-6, IL-12p70, IFN-γ and TNF-α concentrations in the sera were measured in triplicate using standard ELISA kits (Biolegend).

### Real-time PCR

The total RNA was reverse-transcribed into cDNA using Oligo (dT) and M-MLV reverse transcriptase (Promega). The cDNA was subjected to real-time PCR amplification (Qiagen) for 40 cycles with annealing and extension temperature at 60°C, on a LightCycler 480 Real-Time PCR System (Roche). Primer sequences are: : mouse β-Actin forward, 5′-TGGATGACGA TATCGCTGCG-3′; reverse, 5′-AGGGTCAGGATA CCTCTCTT-3′, IL-6 forward, 5′-AACGATGATG CACTTGCAGA-3′; reverse, 5′-GAGCATTGGAAA TTGGGGTA-3′, IL-12p40 forward, 5′-CACATCTGCT GCTCCACAAG-3′; reverse, 5′- CCGTCCGGAGTAA TTTGGTG-3′, IL-23p19 forward, 5′-CTC TCG GAATCTCTGCAT GC-3′; reverse, 5′-ACCATCTTCA CACTGGATACG-3′, TNF-α forward, 5′-CCTTTCAC TCACTGGCCCAA-3′; reverse, 5′-AGTGCCTCTTC TGCCAGTTC-3′ T-bet forward, 5′-CAACAACCCC TTTGCCAAAG-3′; reverse, 5′-TCCCCCAAGCATTG ACAGT-3′, GATA3 forward, 5′-CGGGTTCGGATG TAAGTCGAGG-3′; reverse, 5′- GATGTCCCTGC TCTCCTTGCTG-3′, RORγt forward, 5′-CCGCTGAGA GGGCTTCAC-3′; reverse 5′-TGCAGGAGTAGGCC ACATTACA-3′, IFN-γ forward, 5′-GGATGCATTCA TGAGTATTGC-3′; reverse, 5′-CTTTTCCGCTTC CTGAGG-3′, IL-4 forward, 5′-ACAGGAGAA GGGACGCCAT-3′; reverse 5′-GAAGCCCTACAGA CGAGCTCA-3′, IL-17A forward, 5′-GCGCAAAAGTG AGCTCCAGA-3′; reverse 5′-ACAGAGGGATAT CTATCAGGG-3′.

### OT-I and OT-II T cell proliferation

CD4 T cells from OT-II mice or CD8 T cells from OT-I mice were isolated from spleens using CD4 T cell and CD8 T cell isolation kits (Miltenyi Biotec), respectively. The cells were suspended in PBS/0.1% BSA containing 10 μM CFSE (Invitrogen) for 10 min. CFSE-labeled cells (1 × 10^6^) were *i.v.* transferred into CD45.1 congenic mice, which were treated 24 hours later. At 72 hours after treatment, the splenocytes were harvested and OT-I or OT-II T cell proliferation was determined by analyzing the CFSE fluorescence intensity through flow cytometry.

### Analysis of tumor infiltrated T cells

OT-I or OT-II cells were transferred into B16-OVA tumor-bearing CD45.1 congenic mice; 24 hours later, the mice were injected with PBS, OVA, pullulan, and the combination of OVA and pullulan. Three days after the injection, the tumors were harvested and their weight was measured. The tumors were cut into small fragments and then digested with a 2% fetal bovine serum (FBS) containing collagenase for 20 min at room temperature. The cells from the digest were centrifuged to pellets, which were re-suspended in 4 mL of 1.077 histopaque (Sigma-Aldrich), layered below by an additional 4 ml of histopaque, and then centrifuged at 1700 g for 10 min. The light density fraction (< 1.077 g/cm^3^) was collected and stained with anti-CD45.2, anti-CD3, anti-CD4, and anti-CD8. The infiltrated OT-1 and OT-II cells were defined by CD45.2^+^CD8^+^ or CD45.2^+^CD4^+^ cells in the CD3^+^ cells.

### Intrasplenic injection

C56BL/6 mice were anesthetized using a ketamine mixture (10 μL ketamine HCl, 7.6 μL xylazine, 2.4 μL acepromazine maleate, and 10 μL H_2_O) that was injected into the peritoneal cavity. For the experiments, the B16-OVA melanoma cells (0.5 × 10^6^/50 μL) were inoculated in the spleens of the mice during open laparotomy.

### *In vivo* cytotoxicity assay

As previously described [24], the mice were injected *i.v.* with a mixture of splenocytes labeled with CFSE (20 nM) and loaded with 100 nM SIINFEKL peptide. The spleen cells were labeled using a 10 mM CellTracker^TM^ Orange CMTMR (Life technologies), and they were not loaded with peptide. A total of 10 × 10^6^ cells per mouse were injected, with a mixture containing each target cell population. Splenocytes were collected 6 hours after injection of target cells. The percentage of killing was calculated using the formula described in [35].

### IFN-γ Elispot assay

Splenocytes were isolated 7 days after the last immunization and cultured with 100 ng of SIINFEKL peptides in ELISPOT plates (Nunc) coated with 10 μg/mL rat anti-mouse IFN-γ capture mAbs (Biolegend). 18 hour later, washed plates were incubated with 2 μg/mL biotinylated rat anti-mouse IFN-γ mAbs (Biolegend) followed by the treatment with avidin conjugated alkaline phosphatase (nce) and 50 μL AEC substrate solution (Sigma-Aldrich). Spots were counted using a Bioreader 3000 (Biosys).

### Statistical analysis

The results were expressed as the mean ± standard error of the mean (SEM). The statistical significance of the differences between the experimental groups was calculated using analysis of variance with a Bonferroni post-test or an unpaired Student's t-test. All *p-values < 0.05* were considered significant.
